# Lambs Fed Fresh Winter Forage Rape (*Brassica napus* L.) Emit Less Methane than Those Fed Perennial Ryegrass (*Lolium perenne* L.), and Possible Mechanisms behind the Difference

**DOI:** 10.1371/journal.pone.0119697

**Published:** 2015-03-24

**Authors:** Xuezhao Sun, Gemma Henderson, Faith Cox, German Molano, Scott J. Harrison, Dongwen Luo, Peter H. Janssen, David Pacheco

**Affiliations:** Grasslands Research Centre, AgResearch Limited, Private Bag 11008, Palmerston North, New Zealand; University of Illinois, UNITED STATES

## Abstract

The objectives of this study were to examine long-term effects of feeding forage rape (*Brassica napus* L.) on methane yields (g methane per kg of feed dry matter intake), and to propose mechanisms that may be responsible for lower emissions from lambs fed forage rape compared to perennial ryegrass (*Lolium perenne* L.). The lambs were fed fresh winter forage rape or ryegrass as their sole diet for 15 weeks. Methane yields were measured using open circuit respiration chambers, and were 22-30% smaller from forage rape than from ryegrass (averages of 13.6 g versus 19.5 g after 7 weeks, and 17.8 g versus 22.9 g after 15 weeks). The difference therefore persisted consistently for at least 3 months. The smaller methane yields from forage rape were not related to nitrate or sulfate in the feed, which might act as alternative electron acceptors, or to the levels of the potential inhibitors glucosinolates and S-methyl L-cysteine sulfoxide. Ruminal microbial communities in forage rape-fed lambs were different from those in ryegrass-fed lambs, with greater proportions of potentially propionate-forming bacteria, and were consistent with less hydrogen and hence less methane being produced during fermentation. The molar proportions of ruminal acetate were smaller and those of propionate were greater in forage rape-fed lambs, consistent with the larger propionate-forming populations and less hydrogen production. Forage rape contained more readily fermentable carbohydrates and less structural carbohydrates than ryegrass, and was more rapidly degraded in the rumen, which might favour this fermentation profile. The ruminal pH was lower in forage rape-fed lambs, which might inhibit methanogenic activity, shifting the rumen fermentation to more propionate and less hydrogen and methane. The significance of these two mechanisms remains to be investigated. The results suggest that forage rape is a potential methane mitigation tool in pastoral-based sheep production systems.

## Introduction

Methane (CH_4_) accounts for 37.4% of total anthropogenic greenhouse gas (GHG) emissions in New Zealand [1], and 85% of this is from enteric fermentation in the digestive tracts of grazing ruminants. Enteric CH_4_ is formed mainly in the rumen from hydrogen (H_2_) generated by the rumen microbes when they ferment feed ingested by the animal. Some means to mitigate enteric CH_4_ emissions have been proposed, including manipulation of the rumen microbes using inhibitors or vaccines, modifying the fermentation by supplying H_2_ sinks as feed additives, animal selection for low CH_4_ emitting genotypes, and livestock systems improvement [2–8]. Identifying feeds that result in lower CH_4_ emissions for the same animal production might lead to modified farming systems that have low GHG production. Understanding how low GHG feeds act may also provide opportunities to develop new mitigation technologies, or understand how other potential mitigation tools might perform.

Forage-based mitigation tools would be most easily incorporated into pastoral agriculture by using forage species already accepted or readily incorporated within current systems. Methane emissions from animals fed forage chicory (*Cichorium intybus* L.) or white clover (*Trifolium repens* L.) were not consistently less than from those fed the standard perennial ryegrass diet (*Lolium perenne* L.) [9–14]. In contrast, feeding brassica forages (*Brassica* spp.) resulted in lower CH_4_ emissions from lambs, with the effect being largest for forage rape (*B*. *napus* L.) [15]. Lambs fed forage rape emitted 25% less CH_4_ per unit of dry matter intake compared to ryegrass [15]. However, this result was observed in a single, short term trial only, and no information is available on the persistence of the CH_4_ reduction elicited by feeding forage rape to sheep.

Forage rape has a high nutritional value [15], a high dry matter (DM) yield [16], and supports rapid animal growth [17,18]. Thus, if forage rape fed to ruminants is confirmed to result in lower CH_4_ emissions than ryegrass, and the effect is persistent, this forage would be a practical tool to mitigate CH_4_ as long as it has no negative environmental impacts, such as causing increased emissions of nitrous oxide or nitrogen leaching.

The first objective of this study was to confirm the previous finding [15] that CH_4_ yields (emissions per unit of DM eaten) were smaller when lambs were fed forage rape, and to examine if this effect was stable for a length of time representative of lambs grazing on forage rape in commercial operations. The second objective was to understand how a winter forage rape diet affected *in situ* and *in vivo* digestion and fermentation of the feed, and what its effects were on rumen microbial communities, when compared with perennial ryegrass.

## Materials and Methods

### Ethics Statement

The use of animals, including welfare, husbandry, experimental procedures, and the collection of rumen samples used for this study, was approved by the AgResearch Grasslands (Palmerston North, New Zealand) Animal Ethics Committee (approval numbers 12320 and 12789), and complied with the institutional Codes of Ethical Conduct for the Use of Animals in Research, Testing and Teaching, as prescribed in the Animal Welfare Act of 1999 and its amendments (New Zealand).

### Experimental design

The animal experiment compared CH_4_ emissions from healthy 9-month-old male Romney lambs (*n* = 24) fed fresh winter forage rape (*Brassica napus* L.) with those from lambs (*n* = 18) fed fresh perennial ryegrass (*Lolium perenne* L.) during winter from May to September 2011. Methane emissions and other parameters were determined in two periods (Period 1, days 1–59; Period 2, days 60–117) as described in S1 Text and S1 Table. Details of the experimental animals, forages and feeding, the protocols describing measurement of CH_4_ emissions, digestibility and ME measurements, rumen fluid sampling and sample processing, the determination of rumen liquid and particulate passage rates, *in situ* DM degradation kinetics, methods for determining the nutritional composition of the forages, methods for measuring nitrate, sulfate, glucosinolate and SMCO concentrations, methods for the assessment of rumen microbial community composition, and the statistical analyses used in this study are all described in S1 Text.

## Results

### Methane yields

Lambs were fed either forage rape or ryegrass over two periods, and feed intakes and CH_4_ emissions from individual animals were measured (Table 1). The CH_4_ yield (g/kg DM intake) from the forage rape-fed lambs was 30% smaller (*P*<0.001) than that from the ryegrass-fed lambs. Twice as much H_2_ (g/kg DM intake) was emitted from lambs fed forage rape than from those fed ryegrass (*P* = 0.109). The same animals were maintained on their diets in the second measurement period, and again the CH_4_ yield was smaller, by 22%, from forage rape-fed lambs than for ryegrass-fed ones (*P*<0.001). Compared to Period 1, the H_2_ yield was greater for both diets in Period 2, but the difference between diets was not statistically significant. CH_4_ yields for individual animals were highly correlated between the two periods (r = 0.792, *P*< 0.001; S1 Fig.). The proportion of dietary gross energy lost from the feed as CH_4_ was 21% less for forage rape- than for ryegrass-fed lambs (*P*<0.001; Table 1).

**Table 1:** Methane (CH_4_), hydrogen (H_2_) and carbon dioxide (CO_2_) emissions from lambs fed fresh winter forage rape or fresh perennial ryegrass for 48 h periods in open circuit respiration chambers.

Intakes and emissions	Forage rape	Perennial ryegrass	*P* ^b^
	(*n* = 24)^a^	(*n* = 18)	
**Period 1**			
DM^c^ intake (g/d)	862 ±8.1	792 ±25.9	0.006
CH_4_ (g/d)	11.7 ±0.48	15.4 ±0.97	<0.001
CH_4_ (g/kg DM intake)	13.6 ±0.52	19.5 ±1.14	<0.001
CH_4_ energy loss/gross energy intake	0.050 ±0.0019	0.063 ±0.0039	0.002
CH_4_ (g/LW^d^)	0.316 ±0.0117	0.447 ±0.0139	<0.001
CH_4_ (g/LW^0.75^)	0.843 ±0.0308	1.167 ±0.0367	<0.001
H_2_ (g/kg DM intake)	0.026 ±0.004	0.010 ±0.001	0.109
CO_2_ (g/kg DM intake)	1005 ±6.8	1019 ±22.2	0.480
CH_4_/CO_2_ (mol/mol)	0.039 ±0.0014	0.052 ±0.0030	<0.001
**Period 2**			
DM intake (g/d)	896 ±8.4	929 ±20.8	0.116
CH_4_ (g/d)	16.0 ±0.60	21.2 ±0.50	<0.001
CH_4_ (g/kg DM intake)	17.8 ±0.64	22.9 ±0.45	<0.001
CH_4_ energy loss/gross energy intake	0.058 ±0.0021	0.073 ±0.0014	<0.001
CH_4_ (g/LW^d^)	0.304 ±0.0108	0.392 ±0.0124	<0.001
CH_4_ (g/LW^0.75^)	0.758 ±0.0272	0.981 ±0.0314	<0.001
H_2_ (g/kg DM intake)	0.037 ±0.008	0.033 ±0.006	0.746
CO_2_ (g/kg DM intake)	1190 ±10.7	1065 ±16.3	<0.001
CH_4_/CO_2_ (mol/mol)	0.041 ±0.0014	0.058 ±0.0011	<0.001

^a^ Number of animals sampled. Values are means ± SEM.

^b^ *P* value for the difference between forage rape and perennial ryegrass.

^c^ Dry matter.

^d^ Live weight.

### Forage composition, apparent digestibility and metabolisable energy

The chemical composition of the forage rape and the ryegrass offered to the lambs during the experiment is shown in Table 2. The forage rape contained almost twice the amount of hot water-soluble carbohydrates (*P*≤0.023). The pectin content was also greater in forage rape (*P*<0.001). The amount of readily fermentable carbohydrates in forage rape was 2.37 and 2.35 times that in ryegrass, while the concentrations of structural carbohydrates (NDF and ADF) were much smaller in forage rape than in ryegrass. As a result, the ratio of readily fermentable carbohydrates to structural carbohydrates was much greater in forage rape (1.31 and 2.38 for Periods 1 and 2, respectively) than in ryegrass (0.21 and 0.31, *P*≤0.007).

**Table 2:** Chemical composition of fresh winter forage rape and fresh perennial ryegrass.

Chemical constituent	Forage rape	Perennial ryegrass	*P* ^c^
(g/kg DM^a^ except as noted)	(*n* = 3)^b^	(*n* = 3)	
**Period 1**			
Dry matter (g/kg)^d^	131 ± 2.9	148 ± 4.8	0.005
Organic matter	852 ± 16.4	842 ± 38.4	0.834
Crude protein	215 ± 11.0	181 ± 6.3	0.058
Lipid	34 ± 0.9	41 ± 1.7	0.015
Hot water-soluble carbohydrates	142 ± 11.9	83 ± 11.3	0.023
Pectin	76 ± 2.8	9 ± 0.6	<0.001
Readily fermentable carbohydrates^e^	218 ± 13.9	92 ± 11.8	0.002
NDF^f^	209 ± 17.5	464 ± 23.7	<0.001
ADF^g^	161 ± 16.4	242 ± 26.1	0.059
Hemicellulose	48 ± 1.2	222 ± 5.0	<0.001
Cellulose	124 ± 14.6	215 ± 25.0	0.035
RFC:SC^h^	1.31 ± 0.214	0.21 ± 0.038	0.007
Lignin (sa)^i^	38 ± 9.1	27 ± 1.5	0.323
**Period 2**			
Dry matter (g/kg)	142 ± 2.1	198 ± 4.6	<0.001
Organic matter	917 ± 1.9	901 ± 1.9	0.003
Crude protein	158 ± 2.1	160 ±5.5	0.742
Lipid	34 ± 0.9	35 ± 1.0	0.374
Hot water-soluble carbohydrates	240 ± 2.4	123 ± 8.4	<0.001
Pectin	75 ± 1.7	11 ± 0.3	<0.001
Readily fermentable carbohydrates	315 ± 2.6	134 ± 8.4	<0.001
NDF	170 ± 4.4	445 ± 6.0	<0.001
ADF	123 ± 3.5	231 ± 3.2	<0.001
Hemicellulose	47 ± 0.9	214 ± 2.8	<0.001
Cellulose	86 ± 5.4	214 ± 2.7	<0.001
RFC:SC	2.38 ± 0.120	0.31 ± 0.017	<0.001
Lignin (sa)	37 ± 2.6	17 ± 0.6	0.002

^a^ Dry matter.

^b^ The number of field replicates of forage samples; data are means ± SEM.

^c^ *P* value for the difference between forage rape and perennial ryegrass.

^d^ The number of field replicates of forage samples for the determination of dry matter contents was 18 per forage.

^e^ Hot water-soluble carbohydrates plus pectin.

^f^ Neutral detergent fibre assayed with a heat stable amylase and expressed inclusive of residual ash.

^g^ Acid detergent fibre expressed inclusive of residual ash.

^h^ Ratio of readily fermentable carbohydrates: structural carbohydrates (hemicellulose + cellulose).

^i^ Lignin determined by solubilization of cellulose with sulfuric acid (sa).

The measured apparent digestibility of forage rape in the lambs was greater than that of ryegrass (Table 3). Forage rape had 10–24% greater DM digestibility, and 13–16% greater organic matter and crude protein digestibilities (*P*<0.001). NDF and ADF digestibilities of forage rape were 13–38% greater than those measured from ryegrass in Period 1 (*P*≤0.006), but 10–16% smaller in Period 2 (*P*<0.001).

**Table 3:** Dry matter (DM) intake and apparent total tract digestibility of constituents and energy in lambs fed either fresh winter forage rape or fresh perennial ryegrass.

Digestibility and energy	Forage rape	Perennial ryegrass	*P* ^b^
	(*n* = 6)^a^	(*n* = 6)	
**Period 1**			
DM intake (g/d)	895 ±2.1	826 ±6.4	<0.001
Apparent digestibility (g/kg DM):			
Dry matter	800 ±4.9	646 ±11.3	<0.001
Organic matter	873 ±2.8	751 ±5.3	<0.001
Crude protein	837 ±3.3	736 ±6.2	<0.001
NDF^c^	660 ±15.1	583 ±16.3	0.006
ADF^d^	670 ±14.9	486 ±23.8	<0.001
Energy partition (MJ/kg DM intake):			
Intake gross energy	15.6 ±0.01	16.9 ±0.05	<0.001
Faeces gross energy	2.4 ±0.04	4.2 ±0.08	<0.001
Urine gross energy	0.7 ±0.03	0.9 ±0.02	<0.001
Methane gross energy	0.8 ±0.07	1.0 ±0.04	0.011
DE^e^ (MJ/kg DM intake)	13.2 ±0.04	12.8 ±0.11	<0.001
ME^f^ (MJ/kg DM intake)	11.7 ±0.07	10.8 ±0.11	<0.001
**Period 2**			
DM intake (g/d)	932 ±16.6	1041 ±12.9	<0.001
Apparent digestibility (g/kg DM):			
Dry matter	821 ±2.3	750 ±5.5	<0.001
Organic matter	850 ±2.9	771 ±5.8	<0.001
Crude protein	772 ±6.4	679 ±5.2	<0.001
NDF	632 ±12.4	756 ±5.7	<0.001
ADF	685 ±12.0	764 ±8.8	<0.001
Energy partition (MJ/kg DM intake):			
Intake gross energy	17.2 ±0.04	17.5 ±0.01	<0.001
Faeces gross energy	3.1 ±0.07	4.7 ±0.09	<0.001
Urine gross energy	0.7 ±0.04	0.7 ±0.02	0.259
Methane gross energy	1.0 ±0.08	1.2 ±0.02	0.051
DE (MJ/kg DM intake)	14.1 ±0.04	12.8 ±0.09	<0.001
ME (MJ/kg DM intake)	12.4 ±0.08	10.9 ±0.10	<0.001

^a^ Number of animals sampled. Values are means ± SEM.

^b^ *P* value for the difference between forage rape and perennial ryegrass.

^c^ Neutral detergent fibre assayed with a heat stable amylase and expressed inclusive of residual ash.

^d^ Acid detergent fibre expressed inclusive of residual ash.

^e^ Digestible energy.

^f^ Metabolisable energy.

### *In situ* ruminal DM degradation kinetics

Forage samples collected during the methane and digestibility measurement periods were incubated in the rumen of cows to determine the DM degradation parameters of the two forages. The DM of forage rape had a slightly larger soluble fraction than ryegrass, but a much smaller indigestible fraction than ryegrass in both periods (Table 4; *P*<0.001). The indigestible fraction in forage rape was only 36–39% that of ryegrass. The potentially degradable fraction was similar for the two forages in both periods, but its degradation rate in forage rape was about twice as fast than that of ryegrass (*P*<0.001).

**Table 4:** *In situ* ruminal dry matter degradation kinetics of fresh winter forage rape and fresh perennial ryegrass^a^.

Degradation parameters	Forage rape	Perennial ryegrass	*P* ^c^
	(*n* = 4)^b^	(*n* = 4)	
**Period 1**			
Soluble fraction *A* (g/kg DM^d^)	562 ±10	539 ±9	0.087
Potentially degradable fraction *B* (g/kg DM)	418 ±12.4	403 ±12.4	0.377
Indigestible fraction *C* (g/kg DM)	21 ±4.3	59 ±4.3	<0.001
DM degradation rate *k* (/h)	0.142 ±0.0046	0.071 ±0.0046	<0.001
**Period 2**			
Soluble fraction *A* (g/kg DM)	529 ±9	489 ±9	0.002
Potentially degradable fraction *B* (g/kg DM)	441 ±12.4	434 ±12.4	0.729
Indigestible fraction *C* (g/kg DM)	30 ±4.3	77 ±4.3	<0.001
DM degradation rate *k* (/h)	0.135 ±0.0046	0.077 ±0.0046	<0.001

^a^ *in situ* incubations conducted in the rumens of two cows fed perennial ryegrass. Soluble fraction *A* was calculated from dry matter disappearance at 0 h.

^b^ The number of field replicates of forage samples. Values are means ± SEM.

^c^ *P* value for the difference between forage rape and perennial ryegrass.

^d^ Dry matter.

### Nitrate, sulfate, glucosinolates and SMCO

Forage rape fed to the lambs in Period 1 contained 10 times more nitrate-N than ryegrass (Table 5; *P* = 0.004), but in Period 2 the ryegrass contained more nitrate (15 mmol/kg DM) than the forage rape (where it was below the detection limit of 7.1 mmol/kg DM). Sulfate-S was also higher in forage rape than in ryegrass (*P* = 0.015) in the first period, and the trends were reversed in the second period (*P* = 0.011).

**Table 5:** Nitrate, sulfur and sulfate concentrations in winter forage rape and perennial ryegrass and potential methane reduction from nitrate and sulfate.

Measures	Forage rape	Perennial ryegrass	*P* ^b^
	(*n* = 4)^a^	(*n* = 4)	
**Period 1**			
Total N (mol/kg)	2.52 ±0.061	2.11 ±0.021	<0.001
Nitrate-N (mmol/kg)	135 ±26.0	13 ±3.0	0.004
Total sulfur (mol/kg)	0.15 ±0.010	0.10 ±0.001	0.003
Sulfate (mmol S/kg)	63 ±6.9	39 ±0.9	0.015
CH_4_ (g/kg DM intake)	13.6	19.5	
CH_4_ difference (g/kg DM intake) compared to perennial ryegrass	−5.9		
Potential maximum CH_4_ difference (g/kg DM intake) from nitrate^c^	2.0		
Potential maximum CH_4_ difference (g/kg DM intake) from sulfate^c^	0.4		
Unexplained CH_4_ difference (g/kg DM intake)	−3.5		
**Period 2**			
Nitrogen (mol N/kg)	1.77 ±0.034	1.77 ±0.018	1.000
Nitrate (mmol N/kg)	<7.1^d^	15 ±2.4	0.004
Total sulfur (mol S/kg)	0.10 ±0.002	0.09 ±0.001	0.004
Sulfate (mmol S/kg)	28 ±1.3	34 ±0.8	0.011
CH_4_ (g/kg DM intake)	17.8	22.9	
CH_4_ difference (g/kg DM intake) compared to perennial ryegrass	−5.1		
Potential maximum CH_4_ difference (g/kg DM intake) from nitrate^c^ ^,^ ^d^	−0.1		
Potential maximum CH_4_ difference (g/kg DM intake) from sulfate^c^	−0.1		
Unexplained CH_4_ difference (g/kg DM intake)	−5.3		

^a^ The number of field replicates of forage samples. Values are means ± SEM.

^b^ *P* value for the difference between forage rape and perennial ryegrass.

^c^ The reduction of 1 mol nitrate or 1 mol sulfate uses 4 mol H_2_, which decreases methane formation by 1 mol [19].

^d^ Detection limit for nitrate was 7.1 mmol/kg (100 mg N/kg). For the calculations of CH_4_ reductions and *P* values, it was assumed that nitrate-N was 7.0 mmol/kg.

Forage rape contained greater amounts of glucosinolates and SMCO than ryegrass in both experimental periods (S3 Table). Epiprogoitrin, glucobrassicanapin and glucobrassicin were the major glucosinolates in rape, and the relative proportions of these changed between the two experimental periods.

### Rumen metabolic parameters

Total ruminal VFA concentrations before morning feeding were similar in lambs fed forage rape and ryegrass (*P*>0.05, S4 Table). Feeding resulted in increases in total VFA for both forages, but forage rape resulted in greater total VFA concentrations compared to ryegrass, 2 h after feeding. The proportions of acetate and propionate in total VFA were similar before and after feeding ryegrass (*P*>0.05), but in forage rape-fed animals the proportions of acetate decreased and propionate increased after feeding (*P*<0.001). The ratio of acetate to propionate was smaller (*P*<0.001) for forage rape than for ryegrass before feeding, after feeding, and in the different periods. Butyrate concentrations were similar for both diets prior to feeding, but increased after feeding forage rape and decreased after feeding ryegrass.

**Fig 1:**
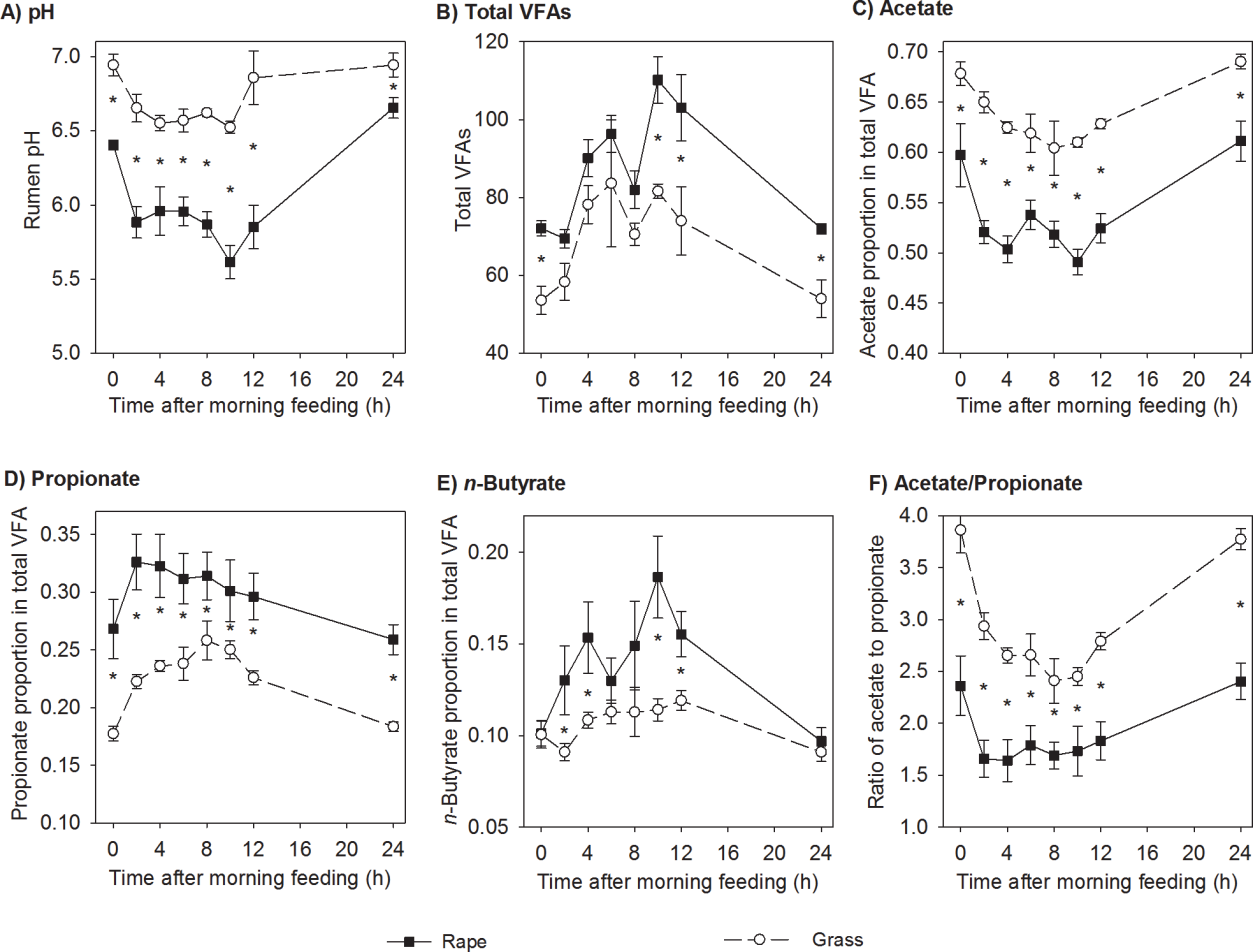
pH (A) and the concentration of total volatile fatty acids (VFAs; B), the molar proportions of individual VFAs (C-E) and the molar ratio of acetate to propionate (F) in the rumen fluid of lambs fed fresh winter forage rape (rape) or fresh perennial ryegrass (grass). The vertical bars indicate one standard error of the mean on either side of the mean.

More intensive sampling was performed during the second measurement period. The rumen pH in the lambs fed forage rape was lower (*P*<0.001) than that in those fed ryegrass at every sampling time, averaging 6.02 for forage rape and 6.71 for ryegrass across 24 h (Fig. 1). The total VFA concentration at each sampling was always greater (*P*<0.001) in the rumens of lambs fed forage rape than for those fed ryegrass. The proportions of acetate in total VFA were smaller (*P*<0.001) and those of propionate (*P*<0.001) and *n*-butyrate (*P*<0.01) were larger for forage rape than for ryegrass. As a result, the ratio of acetate to propionate was smaller for forage rape (1.89) than for ryegrass (2.94).

Lambs fed forage rape or ryegrass had similar rumen liquid volumes (*P* = 0.845), averaging 5.1 L (Table 6). However, the liquid passage rate in the rumen of forage rape fed lambs was almost half (*P*<0.001) of those fed ryegrass. The rumen particulate passage rate was also smaller (by 38%; *P* = 0.029) for forage rape than for ryegrass. As a result, both liquid and particulate fractions had longer retention times in the rumen (*P*<0.05) for forage rape compared with ryegrass.

**Table 6:** Liquid and particulate passage rates and rumen volumes in lambs fed either fresh winter forage rape or fresh perennial ryegrass.

Passage rate parameters	Forage rape	Perennial ryegrass	*P* ^b^
	(*n* = 6)^a^	(*n* = 6)^a^	
**Liquid phase** ^**c**^			
Rumen liquid passage rate *k* (/h)	0.103 ±0.0096	0.193 ±0.0096	<0.001
Liquid retention time (h)	10.4 ±1.24	5.2 ±0.19	0.002
Rumen liquid volume (L)	5.05 ±0.346	5.16 ±0.379	0.845
**Particulate phase** ^**d**^			
First compartment passage rate (*c*_1_, /h)	0.037 ±0.0065	0.060 ±0.0059	0.029
Second compartment passage rate (*c*_2_, /h)	0.046 ±0.0162	0.071 ±0.0162	0.287
Rumen mean retention time (h)	28.3 ±3.19	18.3 ±2.91	0.045
Caecum mean retention time (h)	26.2 ±5.49	22.0 ±5.49	0.609

^a^ The number of animals sampled. Values are means ± SEM.

^b^ *P* value for the difference between forage rape and perennial ryegrass.

^c^ Rumen liquid passage rate and rumen liquid volume were estimated using the method of Faichney [20] with Co-EDTA as the marker.

^d^ Rumen particulate passage rate was estimated using the method of Dhanoa *et al*. [21] with Cr-modanted fibre as the marker.

### Rumen microbial communities

**Fig 2:**
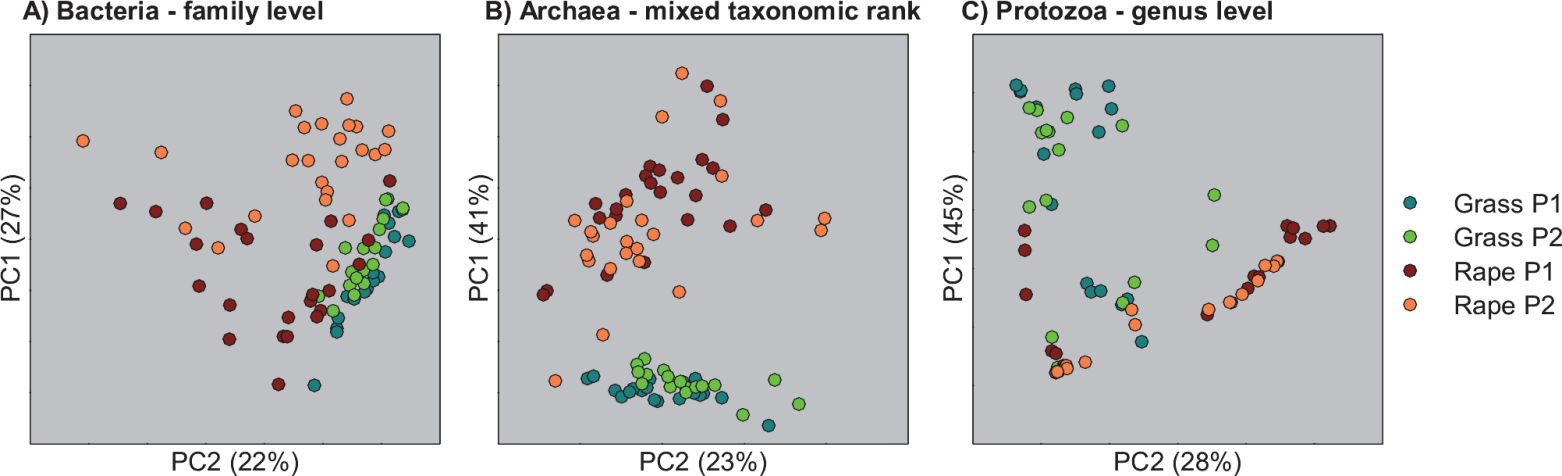
Principal coordinate analysis of Bray-Curtis dissimilarities of bacterial (A), archaeal (B) and protozoal (C) community compositions in the rumen fluid of lambs fed fresh winter forage rape (rape) or fresh perennial ryegrass (grass). The key to the right indicates the different forages and the time period of sampling [Period 1 (P1) or Period 2 (P2)]. The values in parentheses give the amount of variation explained by each coordinate.

**Fig 3:**
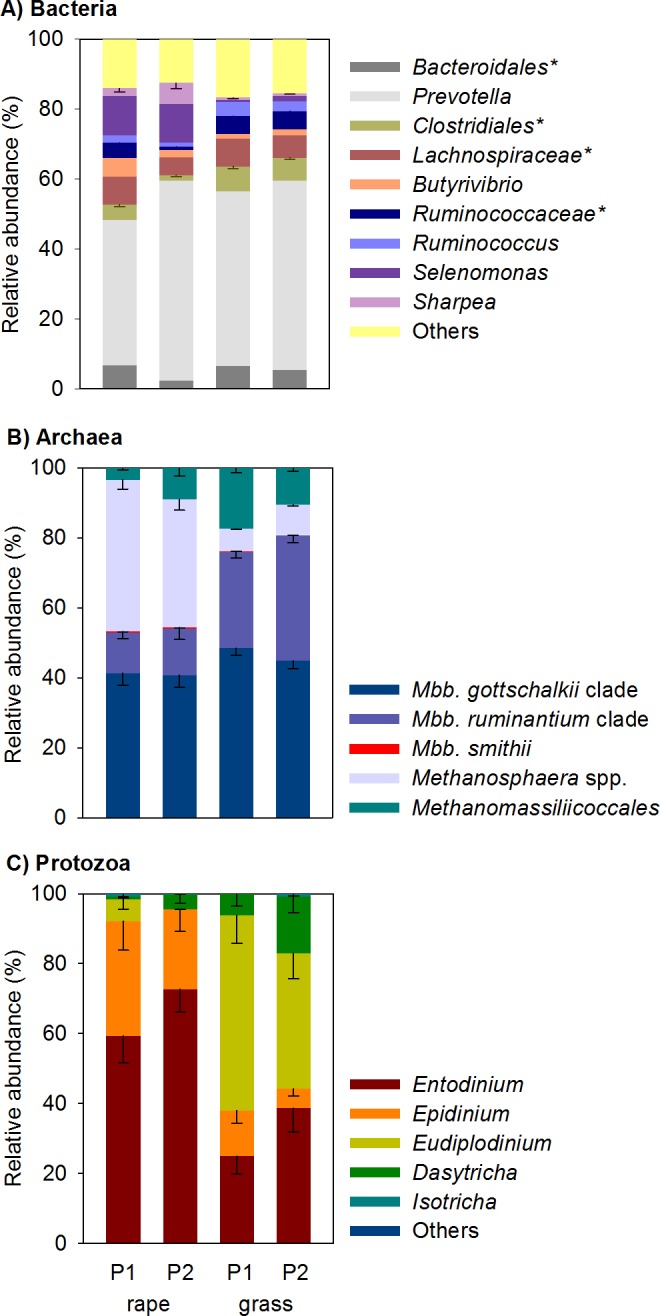
Compositions of the bacterial (A), archaeal (B) and protozoal (C) communities in the rumen fluid of lambs fed fresh winter forage rape (rape) or fresh perennial ryegrass (grass). The key below indicates the different forages and the time period of sampling [Period 1 (P1) or Period 2 (P2)]. The vertical bars indicate one standard error of the mean. Bacteria were analysed at a genus level, and groups labelled * are undefined genera within named higher taxa. Archaea and protozoa were analysed as in Fig. 2. More details of the bacterial community can be found in S5 Table.

Bacterial, archaeal, and protozoal microbial community compositions were compared between forages and measurement periods. There was little similarity of bacterial, archaeal or protozoal communities of forage rape and ryegrass-fed animals in principal coordinate analyses, indicating that their rumen microbial communities were different between diets (Fig. 2). Interestingly, the data points for the rumen archaeal and bacterial communities of forage rape-fed animals were more widely spread than those of ryegrass-fed animals, whereas protozoal community data points were more widely spread in ryegrass-fed animals. A shift in the rumen bacterial community composition of the forage rape-fed lambs was evident between measurement periods (Figs. 2 and 3). A subtle shift was also detected in the relative abundances within the methanogen community in the rumens of animals fed perennial ryegrass in the two periods (Fig. 3), which was too small to see clearly by principal coordinate analysis (Fig. 2).

The main underlying differences in the microbial community composition of forage rape-fed sheep relative to ryegrass-fed animals were greater relative abundances (*P*<0.001 unless noted otherwise) of sequences assigned to the genera *Selenomonas*, *Butyrivibrio*, *Sharpea* (*P* = 0.004), and *Methanosphaera*, and lower relative abundances of members of the *Methanobrevibacter ruminantium* clade, *Eudiplodinium*, *Oscillospira*, undefined genera affiliated with *Ruminococcaceae*, undefined genera affiliated with *Clostridiales*, and undefined genera affiliated with candidate division TM7.

Total bacterial and archaeal marker gene copy numbers determined using quantitative PCR showed that the ratio of archaea to bacteria was greater in rumen samples from ryegrass-fed lambs (4.6 × 10^9^ ± 5.7 × 10^8^ archaea and 7.5 × 10^11^ ± 9.2 × 10^10^ bacteria g^-1^ dry weight rumen contents, 0.0063 ± 0.0006 archaea:bacteria) than in rape-fed lambs (4.2 × 10^9^ ± 6.0 × 10^8^ archaea and 1.1 × 10^12^ ± 8.2 × 10^10^ bacteria g^-1^ dry weight rumen contents, 0.0040 ± 0.0005; *P* = 0.012). Protozoal cell numbers were significantly smaller in the rumens of lambs fed ryegrass than those fed forage rape (S6 Table).

The decrease in CH_4_ yield in forage rape-fed animals was strongly correlated with an increase in *Methanosphaera* (r = −0.777, *P*<0.001). Acetate (as a proportion of total volatile fatty acids) correlated negatively with the relative abundance of *Methanosphaera* (r = −0.781, *P*<0.001), but positively with the relative abundance of *Ruminococcus* (r = 0.626, *P*<0.001). Propionate was positively correlated with the relative abundance of *Selenomonas* and relatives (r = 0.762, *P*<0.001). Butyrate levels and the relative abundance of *Butyrivibrio* were also correlated (r = 0.622, *P*<0.001).

## Discussion

### Methane emissions from forage rape

In this study, lambs fed fresh winter forage rape for 7 and 15 weeks emitted 30% and 22% less CH_4_ per unit of feed eaten, respectively, than lambs fed perennial ryegrass. These results confirmed our earlier findings [15], that CH_4_ emissions from lambs fed forage rape were 25% smaller compared to ryegrass. The results from the present study indicated that differences in CH_4_ emissions were long lasting when the forage rape was continuously fed. The differences in CH_4_ yield were not the same at 7 and 15 weeks, which may be due to seasonal effects on the animals or changes in the characteristics of the forages. In commercial operations, forage rape is often used as a finishing diet for about three months, and the differences in CH_4_ emissions persisted over this length of time.

### Volatile fatty acids

In both measurement periods of the experiment, lambs fed forage rape had greater molar proportions of propionate and smaller molar proportions of acetate in the rumen than those that ate ryegrass. This result is consistent with our previous finding [15]. Propionate formation from carbohydrates is an electron-consuming process, whereas acetate formation is an electron-producing one. Excess electrons can be disposed of by H_2_ formation by the fermenting bacteria. Therefore, increased propionate formation is associated with less H_2_ formation, and so with less CH_4_ production [22]. The smaller ratio of acetate to propionate in the present study, and so presumably less H_2_ formation, could be a reason for reduced CH_4_ emissions from forage rape. Propionate formation is expected to be favoured by larger ruminal H_2_ concentrations [22], consistent with the higher levels of H_2_ escape measured from the rumen of lambs fed forage rape.

### Rumen microbial communities

The greater ratio of readily fermentable to structural carbohydrates in forage rape compared to ryegrass may result in greater feed digestibility and a larger degradation rate, and in a lower ruminal pH which is suboptimal for methanogens. These changes are postulated to increase local H_2_ concentrations and increase propionate formation, resulting in overall less H_2_ and CH_4_ being formed [22]. The differences observed between the microbial communities of sheep fed forage rape and ryegrass fitted with this conceptual model. The communities in sheep fed forage rape were similar to those previously found in animals fed a high-grain diet [23,24]. For example, compared to ryegrass-fed sheep, propionate, butyrate and total VFA concentrations were greater in the rumens of sheep fed forage rape, as were the relative abundances of *Selenomonas* spp. and their relatives, which produce propionate, and of *Butyrivibrio* spp., which produce butyrate, as major fermentation end products. Closer inspection of the 16S rRNA gene sequences affiliated with *Selenomonas* spp. revealed that many could belong to the poorly-studied genus *Quinella*. The greater abundance of these genera may in part be due to the larger concentrations of readily fermentable carbohydrates present in forage rape. The relative abundance of the genus *Sharpea* was also greater in forage rape-fed animals. Members of the genus *Sharpea* (which includes the species *Kandleria vitulina* [25]) are able to tolerate low pH [26,27]. It is also noteworthy that these same bacterial groups are associated with naturally low CH_4_ emissions from sheep [28].

The abundance of *Oscillospira* has previously been reported to be smaller in grain-fed animals compared to animals on pasture [29]. The rape-fed lambs in our study also had lower abundances of *Oscillospira*, indicating that rumen bacterial communities of rape and grain-fed animals may share some characteristics. The lower relative abundance of fibre- and cellulose-degrading bacteria such as *Fibrobacter* spp. and undefined genera within the family *Ruminococcaceae* in forage rape-fed animals is likely linked to the smaller concentration of structural carbohydrates (NDF, ADF, [hemi-]cellulose) present in forage rape, which in turn may result in less acetate and H_2_ being formed. Consistent with less H_2_ formation during feed fermentation, methanogens made up a smaller proportion of the rumen microbial community, relative to the bacteria, in lambs fed forage rape than in those fed ryegrass. Less H_2_ formation would support smaller populations of methanogens.

Forage rape was also found to contain greater levels of pectin than ryegrass. Bacteria [30] and protozoa [31] are able to release methanol from pectin. *Methanosphaera* spp. reduce 1 mol of methanol with 1 mol of H_2_ to generate CH_4_ [32]. In contrast, *Methanobrevibacter* spp. reduce CO_2_ with 4 H_2_ to produce CH_4_. The increased availability of methanol in the pectin-rich rape diet favours *Methanosphaera* spp., while the shift away from H_2_ production and towards propionate production in the feed fermentation reduces the population of *Methanobrevibacter* spp. Together, this likely explains the increased significance of *Methanosphaera* spp. in the forage rape-fed lambs and the negative correlation of *Methanosphaera* with CH_4_.

### Potential mechanisms for lower methane emissions from forage rape

The conventional chemical composition of forage rape (Table 2) was markedly different to that of ryegrass, with more readily fermentable and less structural carbohydrates in forage rape than in ryegrass. Forage chicory [11,12], white clover [13,14] and a range of other forage brassicas [15] also contained more readily fermentable carbohydrates than ryegrass, but forage chicory [11,12] and white clover [13,14] did not result in smaller CH_4_ emissions while other forage brassicas did [15]. Thus it seems that some element of brassica composition, not captured in routine nutritional analysis of animal feed, results in smaller CH_4_ emissions.

Nitrate and sulfate can be electron sinks for anaerobic microbes. Their use as electron acceptors diverts electrons from H_2_ formation, and so from CH_4_ formation in the rumen [19]. The maximum potential CH_4_ reductions attributable to these sinks were estimated (Table 5). Because 1 mol nitrate or 1 mol sulfate use the same amount of hydrogen as consumed in 1 mol CH_4_ formation [19], in the first period, maximally 41% of the smaller CH_4_ emissions could be explained by the reduction of nitrate and sulfate, although their real contribution to the mitigation of CH_4_ emissions was not directly measured. In the second period, nitrate and sulfate concentrations were larger in the ryegrass than in the forage rape, and CH_4_ formation was still smaller from the forage rape-fed lambs. We conclude that the differences in CH_4_ emissions were not driven by nitrate and sulfate in the feeds.

The forage rape diet contained more glucosinolates and SMCO than did the ryegrass. However, in our previous study [15], both glucosinolates and SMCO were not associated with CH_4_ yields. In the present study, total glucosinolate and SMCO levels in the forage rape increased between the first and second periods, but the CH_4_ yield was not reduced. There were changes in the methanogen community composition, and these may be due to a change in the rumen fermentation or pH, or due to a replacement of methanogens more sensitive to these plant secondary metabolites by other that are less sensitive. There may have been effects by inhibitors on methanogen species composition or on the primary fermentation, but these effects could not be assessed using our experimental design. Of significance is the observation that CH_4_ formation was not lower from forage brassicas than from ryegrass when fermented *in vitro* using rumen contents from ryegrass-fed animals (X. Sun *et al*., unpublished data), suggesting that components of forage rape do not inhibit methanogens. Instead it is more likely that forage rape-fed lambs have a different microbial community that produces less H_2_ and less CH_4_, an effect that cannot be detected in the short-term *in vitro* fermentations.

Both liquid and particulate passage rates were slower in forage rape-fed lambs than in lambs fed ryegrass. This finding is consistent with those of Huhtanen and Jaakkola [33], who fed bulls barn-dried grass or direct-cut silage with different proportions of concentrates, and found that the passage rate decreased with the increase of the rapidly-degradable fraction (concentrates) in the diet. Our findings contrast those of Hammond *et al*. [34], who found that increased feeding levels decreased CH_4_ yield, but increased both rumen liquid and solid passage rates when sheep were fed fresh perennial ryegrass. Our findings are also different from those of Goopy *et al*. [35], who compared naturally low and high CH_4_ emitting sheep on a single diet and found that lower emitters had greater particulate and liquid passage rates. Forage rape had a faster fractional degradation rate in the rumen, which may result from larger contents of readily fermentable carbohydrates in the forage. Although the particulate passage rate was smaller than that of DM degradation rate, the overall rate of DM disappearance from the rumen (particular passage rate plus DM degradation rate) was still greater for forage rape (0.179/h) than for ryegrass (0.131/h). The results suggest that the CH_4_ effects are due to the greater rate of forage rape degradation in the rumen rather than to increased passage rates, compared to ryegrass. The rapid fermentation might cause increases in dissolved H_2_, resulting in a shift of rumen fermentation pattern towards less acetate and H_2_ and more propionate being produced and finally to less CH_4_ being formed [22]. Although ruminal H_2_ concentrations were not measured, there was a trend to greater H_2_ emissions from the forage rape-fed sheep, which suggests greater ruminal H_2_ concentrations. These data have to be treated cautiously, as the H_2_ measurements in the respiration chambers are close to the lower limits of detection [36]. However, greater H_2_ emissions were also seen from other brassica feeds that resulted in lower CH_4_ yields [15]. It should be noted that the H_2_ emissions can only account for 0.01 to 1.1% of the CH_4_ differences in this and our earlier study. Our finding are therefore consistent with a change in ruminal H_2_ concentrations that might have an effect on fermentation patterns [22] rather than inhibition of CH_4_ formation from H_2_ with subsequent emission of H_2_ instead of CH_4_.

## Conclusions

CH_4_ yields from lambs fed fresh winter forage rape were 22–30% smaller than those fed perennial ryegrass and the difference persisted for 15 weeks. The lower CH_4_ yields from forage rape are associated with a different rumen fermentation profile, *i*.*e*. lower ratio of acetate to propionate from forage rape than from ryegrass, with lower ruminal pH, and with very different rumen microbial communities. The differences in fermentation pattern appear to be driven by characteristics of the feed, such as the rate of degradation in the rumen and the presence of more readily fermentable carbohydrates, compared to ryegrass. The rapid fermentation may select for the different microbial community directly, or it may do so as a result of the lower ruminal pH. The relative contributions of these two potential mechanisms remain to be determined. From the results presented, we conclude that forage rape could be a viable CH_4_ mitigation tool for pastoral-based sheep production systems.

Although this and previous [15] studies both indicated feeding forage rape results in lower CH_4_ emissions than does ryegrass, these studies were conducted indoors. Before translating these effects to practical farming conditions, it will be necessary to assess the results under conditions that are representative of grazing conditions, as animal behaviour and eating patterns may differ between indoor housing and outdoor grazing. In addition, nitrous oxide emissions from animal excreta and soil cultivation should be included for an integrated evaluation on total greenhouse gas emissions.

## Supporting Information

S1 Fig CH_4_ yields from lambs fed either fresh winter forage rape (rape) or fresh perennial ryegrass (grass). The yields from the two measurement periods are plotted so that each individual lamb is represented as one point. The formula is the regression of Period 1 against Period 2.
(DOCX)

S1 Table Experimental design, showing the allocation of animals to experimental groups, and their locations and the experiments being performed over the course of the trial (DOCX)

S2 Table Blood parameters of lambs fed fresh winter forage rape or fresh perennial ryegrass. (DOCX)

S3 Table Relative concentrations of glucosinolates and *S*-methyl L-cysteine sulfoxide (SMCO) in winter forage rape and perennial ryegrass fed to lambs during the methane measurement period. (DOCX)

S4 Table The concentration of total volatile fatty acids (VFA), the molar proportions of individual VFAs and the ratio of acetate to propionate in the rumen fluid of lambs fed fresh winter forage rape or fresh perennial ryegrass. (DOCX)

S5 Table Effects of forage and experimental period (P1 and P2) on the apparent microbial community structure in the rumens of lambs fed fresh winter forage rape or fresh perennial ryegrass. This is supplied as an XLSX file.
(XLSX)

S6 Table Effects of forage and sampling time on protozoal cell densities in the rumens of lambs fed fresh winter forage rape or fresh perennial ryegrass. (DOCX)

S1 Text Supporting Materials and Methods and Supporting Results. (DOCX)
