# Supplementary material for: Lambs Fed Fresh Winter Forage Rape (Brassica napus L.) Emit Less Methane than Those Fed Perennial Ryegrass (Lolium perenne L.), and Possible Mechanisms behind the Difference
Source: PLoS One. 2015 Mar 24;10(3):e0119697. doi: 10.1371/journal.pone.0119697 (PMC4372518; doi:10.1371/journal.pone.0119697)
Supplement: S1 Fig — The yields from the two measurement periods are plotted so that each individual lamb is represented as one point. The formula is the regression of Period 1 against Period 2. (DOCX) [file pone.0119697.s001.docx]

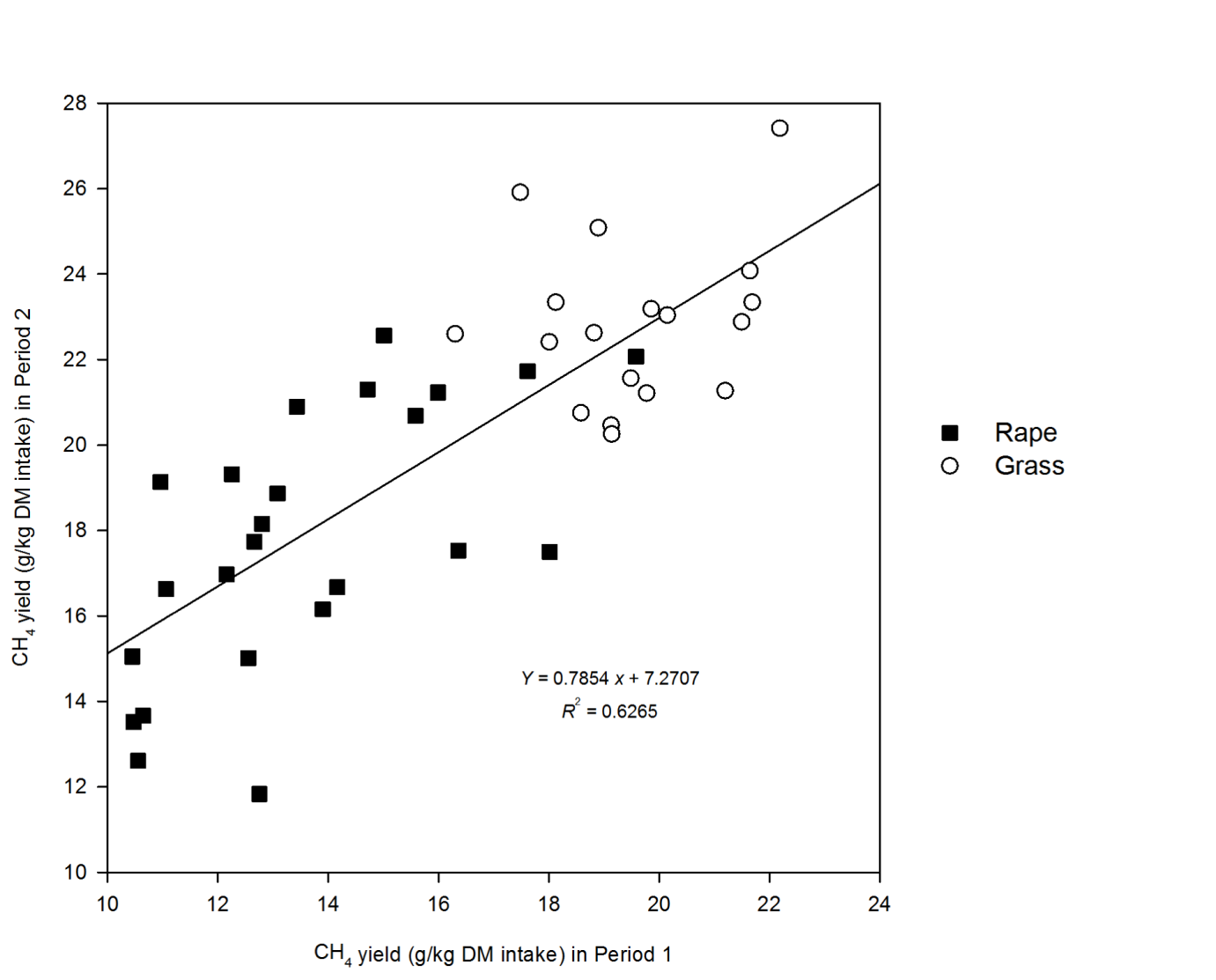


**Figure S1. CH_4_ yields from lambs fed either fresh winter forage rape (rape) or fresh perennial ryegrass (grass).** The yields from the two measurement periods are plotted so that each individual lamb is represented as one point. The formula is the regression of Period 1 against Period 2.
