# Supplementary material for: Lambs Fed Fresh Winter Forage Rape (Brassica napus L.) Emit Less Methane than Those Fed Perennial Ryegrass (Lolium perenne L.), and Possible Mechanisms behind the Difference
Source: PLoS One. 2015 Mar 24;10(3):e0119697. doi: 10.1371/journal.pone.0119697 (PMC4372518; doi:10.1371/journal.pone.0119697)
Supplement: S1 Table — (DOCX) [file pone.0119697.s002.docx]

**Table S1.** Experimental design, showing the allocation of animals to experimental groups, and their locations and the experiments being performed over the course of the trial.

| **Days** | **Period** | **Animals, location and activity** | | |
| --- | --- | --- | --- | --- |
| **1^a^-41** |  | All animals in paddocks for adaptation: 24 on forage rape, 18 on ryegrass (Maintained on the same forages through to end of trial). | | |
| **42-44** |  | All animals in pens for adaptation. | | |
| **45** |  | All animals in crates for adaptation. | | |
| **46** |  | All animals in crates for adaptation. Rumen sampling after morning feeding. Blood samples from 5 randomly-selected animals in the rape group and 3 in the ryegrass group for methaemoglobin determination, and from all animals for haemoglobin and haematocrit determinations. | | |
| **47-49** |  | Random allocation of animals to batch 1 (12 from rape group, 9 from ryegrass group). In crates for adaptation. | | Random allocation of animals to batch 2 (12 from rape group, 9 from ryegrass group). In crates for adaptation. |
| **50-51** | 1 | Batch 1 animals in chambers for CH_4_ measurements. Rumen sampling before feeding after CH_4_ measurements were completed. | |  |
| **52** |  | Allocation of animals (6 on rape and 6 on ryegrass) from batch 1 for digestibility measurements. In crates for adaptation. | Remaining 9 animals from batch 1 in pens. | Batch 2 animals in chambers for CH_4_ measurements. Rumen sampling before feeding after CH_4_ measurements were completed. |
| **53** |  |  |  |  |
| **54-59** |  | 12 allocated animals in crates for digestibility measurements. | Animals in paddocks on appropriate forage. | |
| **60-97** |  |  |  | |
| **98-100** |  | All animals in pens for adaptation. | | |
| **101** |  | All animals in crates for adaptation. | | |
| **102** |  | All animals in crates for adaptation. Rumen sampling from all animals after morning feeding. Blood samples from 6 randomly-selected animals in the rape group and 4 in the ryegrass group for methaemoglobin determination, and from all animals for haemoglobin and haematocrit determinations. | | |
| **103-104** |  | Random allocation of animals to batch 1 (12 from rape group, 9 from ryegrass group). In crates for adaptation. | | Random allocation of animals to batch 2 (12 from rape group, 9 from ryegrass group). In crates for adaptation. |
| **105-106** | 2 | Batch 1 animals in chambers for CH_4_ measurements. | |  |
| **107** |  | Allocation of animals (6 on rape and 6 on ryegrass) from batch 1 for turnover and digestibility measurements. In crates for adaptation. | Remaining 9 animals from batch 1 in pens. | Batch 2 animals in chambers for CH_4_ measurements. |
| **108** |  |  |  |  |
| **109-116** |  | 12 allocated animals in crates for turnover and digestibility measurements. |  | |
| **117** |  | Release of animals to paddock (ryegrass/white clover pasture). | | |

^a^ Day 1 was 17 May 2011.
