# Supplementary material for: Lambs Fed Fresh Winter Forage Rape (Brassica napus L.) Emit Less Methane than Those Fed Perennial Ryegrass (Lolium perenne L.), and Possible Mechanisms behind the Difference
Source: PLoS One. 2015 Mar 24;10(3):e0119697. doi: 10.1371/journal.pone.0119697 (PMC4372518; doi:10.1371/journal.pone.0119697)
Supplement: S2 Table — (DOCX) [file pone.0119697.s003.docx]

**Table S2.** Blood parameters of lambs fed fresh winter forage rape or fresh perennial ryegrass.

| **Blood parameters** | **Forage rape (*n*=24)^a^** | **Perennial ryegrass (*n*=18)** | **Normal range** | **Pooled SEM** | ***P*^b^** |
| --- | --- | --- | --- | --- | --- |
| **Period 1** |  |  |  |  |  |
| White blood cells (WBC, 10^9^/L) | 7.7 | 9.9 | 4.0 - 12.0 | 0.56 | <0.001 |
| Red blood cells (RBC, 10^12^/L) | 9.6 | 12.6 | 9.0 - 15.0 | 0.31 | <0.001 |
| Haemoglobin (Hb, g/L) | 109 | 127 | 90 - 150 | 2.72 | <0.001 |
| Hematocrit (Hct, L/L) | 0.31 | 0.34 | 0.27 - 0.45 | 0.011 | <0.001 |
| Mean corpuscular volume (MCV, fL) | 33 | 27 | 28 - 40 | 0.64 | <0.001 |
| Mean corpuscular haemoglobin (MCH, pg) | 11 | 10 | 8 - 13 | 0.24 | <0.001 |
| Mean corpuscular hemoglobin concentration (MCHC, g/L) | 352 | 370 | 310 - 360 | 2.42 | <0.001 |
| Methaemoglobin (MetHb, %) | 5.5 (*n*=5)^a^ | 1.0 (*n*=3) | <10 | 0.49 | <0.001 |
|  |  |  |  |  |  |
| **Period 2** |  |  |  |  |  |
| WBC (10^9^/L) | 8.0 | 10.2 | 4.0 - 12.0 | 0.54 | <0.001 |
| RBC (10^12^/L) | 12.3 | 12.8 | 9.0 - 15.0 | 0.39 | 0.201 |
| Hb (g/L) | 121 | 123 | 90 - 150 | 3.5 | 0.722 |
| Hct (L/L) | 0.33 | 0.33 | 0.27 - 0.45 | 0.011 | 0.853 |
| MCV (fL) | 27 | 26 | 28 - 40 | 0.4 | 0.007 |
| MCH (pg) | 10 | 10 | 8 - 13 | 0.2 | 0.246 |
| MCHC (g/L) | 368 | 375 | 310 - 360 | 3.1 | 0.054 |
| Tissue plasminogen activator PLAT (10^9^/L) | 580 | 439 | 250 - 750 | 51.2 | 0.009 |
| MetHb (%) | 3.2 (*n*=6) | 1.2 (*n*=4) | <10 | 0.51 | 0.005 |

^a^ Number of animals sampled, unless noted otherwise.

^b^ *P* value for the difference between forage rape and perennial ryegrass.
