# Supplementary material for: Lambs Fed Fresh Winter Forage Rape (Brassica napus L.) Emit Less Methane than Those Fed Perennial Ryegrass (Lolium perenne L.), and Possible Mechanisms behind the Difference
Source: PLoS One. 2015 Mar 24;10(3):e0119697. doi: 10.1371/journal.pone.0119697 (PMC4372518; doi:10.1371/journal.pone.0119697)
Supplement: S3 Table — (DOCX) [file pone.0119697.s004.docx]

Table S3. Relative concentrations of glucosinolates and *S*-methyl L-cysteine sulfoxide (SMCO) in winter forage rape and perennial ryegrass fed to lambs during the methane measurement period.

|  | Period 1 |  |  | Period 2 |  |
| --- | --- | --- | --- | --- | --- |
| Glucosinolates^a^ | Forage rape (*n*=6)^b^ | Perennial ryegrass (*n*=6) |  | Forage rape (*n*=4) | Perennial ryegrass (*n*=4) |
| Gluconasturtiin | 3.6^c^ | 0.0 |  | 4.4 | 0.0 |
| Glucobrassicanapin | 13.1 | 0.0 |  | 30.6 | 0.0 |
| Gluconapin | 3.4 | 0.0 |  | 7.4 | 0.0 |
| Gluconapoleiferin | 5.1 | 0.0 |  | 7.2 | 0.0 |
| Glucoalyssin | 1.9 | 0.0 |  | 4.3 | 0.0 |
| Glucobrassicin | 10.2 | 0.0 |  | 4.1 | 0.0 |
| Epiprogoitrin | 24.0 | 0.0 |  | 40.1 | 0.0 |
| Glucoraphanin | 0.7 | 0.0 |  | 1.8 | 0.0 |
| 4-hydroxyglucobrassicin | 0.0 | 0.0 |  | 0.1 | 0.0 |
| Total glucosinolates | 62.1 | 0.1 |  | 100.0 | 0.0 |
| SMCO | 94.7^d^ | 0.2 |  | 100.0 | 0.0 |

^a^ The following glucosinolates were not detected in any samples: glucoerucin, glucoiberin, progoitrin, sinalbin, glucotropaeolin, glucobarbarin, sinigrin, glucosibarin, or glucoraphenin. The methods used would have detected them.

^b^ The number of field replicates of forage samples.

^c^ Relative value with the total concentration of glucosinolates in forage rape in Period 2 as 100.

^d^ Relative value with SMCO concentration in forage rape in Period 2 as 100.
