# Supplementary material for: Lambs Fed Fresh Winter Forage Rape (Brassica napus L.) Emit Less Methane than Those Fed Perennial Ryegrass (Lolium perenne L.), and Possible Mechanisms behind the Difference
Source: PLoS One. 2015 Mar 24;10(3):e0119697. doi: 10.1371/journal.pone.0119697 (PMC4372518; doi:10.1371/journal.pone.0119697)
Supplement: S4 Table — (DOCX) [file pone.0119697.s005.docx]

**Table S4.** The concentration of total volatile fatty acids (VFA), the molar proportions of individual VFAs and the ratio of acetate to propionate in the rumen fluid of lambs fed fresh winter forage rape or fresh perennial ryegrass.

| **VFA** | **Forage rape** | | | |  | **Perennial ryegrass** | | | |  | ***P*** | | |
| --- | --- | --- | --- | --- | --- | --- | --- | --- | --- | --- | --- | --- | --- |
|  | **Pre-feeding^a^** | | **Post-feeding^a^** | |  | **Pre-feeding^a^** | | **Post-feeding^a^** | |  | **Forage** | **Time^b^** | **Forage× Time** |
| **Period 1^c^** |  | |  |  |  |  | |  | |  |  |  |  |
| Total VFA (mM) | 51.2 | ±2.65 | 104.1 | ±2.70 |  | 51.3 | ±3.15 | 74.5 | ±3.06 |  | <0.001 | <0.001 | <0.001 |
| VFA proportion |  |  |  |  |  |  |  |  |  |  |  |  |  |
| Acetate | 0.600 | ±0.0072 | 0.534 | ±0.0074 |  | 0.677 | ±0.0086 | 0.679 | ±0.0084 |  | <0.001 | <0.001 | <0.001 |
| Propionate | 0.224 | ±0.0065 | 0.310 | ±0.0066 |  | 0.176 | ±0.0077 | 0.194 | ±0.0074 |  | <0.001 | <0.001 | <0.001 |
| *n*-butyrate | 0.100 | ±0.0040 | 0.125 | ±0.0041 |  | 0.094 | ±0.0047 | 0.086 | ±0.0046 |  | <0.001 | 0.010 | <0.001 |
| Minor VFA^d^ | 0.076 | ±0.0030 | 0.031 | ±0.0030 |  | 0.053 | ±0.0035 | 0.040 | ±0.0034 |  | 0.040 | <0.001 | <0.001 |
| Acetate:propionate ratio | 2.75 | ±0.094 | 1.77 | ±0.096 |  | 3.88 | ±0.111 | 3.53 | ±0.108 |  | <0.001 | <0.001 | 0.003 |
|  |  |  |  |  |  |  |  |  |  |  |  |  |  |
| **Period 2 ^c^** |  |  |  |  |  |  |  |  |  |  |  |  |  |
| Total VFA (mM) | 72.1 | ±1.93 | 69.5 | ±2.39 |  | 53.6 | ±3.64 | 58.3 | ±4.75 |  | 0.002 | 0.730 | 0.247 |
| VFA proportion |  |  |  |  |  |  |  |  |  |  |  |  |  |
| Acetate | 0.597 | ±0.0313 | 0.520 | ±0.0114 |  | 0.678 | ±0.0117 | 0.650 | ±0.0103 |  | <0.001 | 0.002 | 0.083 |
| Propionate | 0.268 | ±0.0256 | 0.326 | ±0.0243 |  | 0.177 | ±0.0064 | 0.223 | ±0.0061 |  | <0.001 | 0.003 | 0.798 |
| *n*-butyrate | 0.101 | ±0.0069 | 0.130 | ±0.0187 |  | 0.100 | ±0.0074 | 0.091 | ±0.0047 |  | 0.115 | 0.362 | 0.089 |
| Minor VFA | 0.033 | ±0.0031 | 0.023 | ±0.0015 |  | 0.044 | ±0.0026 | 0.036 | ±0.0022 |  | 0.004 | <0.001 | 0.264 |
| Acetate:propionate ratio | 2.36 | ±0.287 | 1.66 | ±0.177 |  | 3.86 | ±0.218 | 2.93 | ±0.129 |  | <0.001 | 0.001 | 0.544 |

^a^ All values are means ± SEM.

^b^ Sampling time.

^c^ Number of animals sampled in Period 1: n=24 for forage rape, 18 for perennial ryegrass; Period 2: n=6 for forage rape, 6 for perennial ryegrass.

^d^ Minor VFA are *iso*-butyrate, *iso*-valerate, 2-methylbutyrate, and *n*-valerate.
