# Supplementary material for: Lambs Fed Fresh Winter Forage Rape (Brassica napus L.) Emit Less Methane than Those Fed Perennial Ryegrass (Lolium perenne L.), and Possible Mechanisms behind the Difference
Source: PLoS One. 2015 Mar 24;10(3):e0119697. doi: 10.1371/journal.pone.0119697 (PMC4372518; doi:10.1371/journal.pone.0119697)
Supplement: S6 Table — (DOCX) [file pone.0119697.s007.docx]

**Table S6.** Effects of forage and sampling time on protozoal cell densities in the rumens of lambs fed fresh winter forage rape or fresh perennial ryegrass*^a^*

Densities were determined by counting using microscopy. Protozoal cell density data were analysed using two-way ANOVA with forage and sampling time as factors.

| **Forage** | **Sampling time** | **Count ± SEM^b^** |
| --- | --- | --- |
|  |  | **(10^5^ cells/ml)** |
| Forage rape | Pre-feeding (*n*=13)^c^ | 3.3 ± 0.40 |
|  | Post-feeding (*n*=24) | 1.9 ± 0.14 |
| Ryegrass | Pre-feeding (*n*=10) | 2.2 ± 0.26 |
|  | Post-feeding (*n*=18) | 1.4 ± 0.14 |

^a^ Significance of differences were *P* = 0.016 for forge, *P* <0.001 for sampling time, and *P* = 0.327 for forage × sampling time.

^b^ Standard error of the mean.

^c^ Number of animals sampled.
