# Supplementary material for: Lambs Fed Fresh Winter Forage Rape (Brassica napus L.) Emit Less Methane than Those Fed Perennial Ryegrass (Lolium perenne L.), and Possible Mechanisms behind the Difference
Source: PLoS One. 2015 Mar 24;10(3):e0119697. doi: 10.1371/journal.pone.0119697 (PMC4372518; doi:10.1371/journal.pone.0119697)
Supplement: S1 Text — (DOCX) [file pone.0119697.s008.docx]

**Text S1**

**Detailed Materials and Methods**

**Animals and experimental design**

The animal experiment compared methane (CH_4_) emissions from lambs fed fresh winter forage rape (*Brassica napus* L.) with those fed fresh perennial ryegrass (*Lolium perenne* L.). It was conducted at AgResearch Aorangi Experimental Station (for acclimatisation and maintenance of the animals on the diets in paddocks) and at AgResearch Grasslands Research Centre (for indoor measurements), Palmerston North, New Zealand. The experimental sequence is shown in Table S1. During the experiment, lambs were treated and monitored for health status and welfare.

Sixty growing lambs with similar live weights [32.4 ±0.6 kg, mean ± S.D., on day 1 (17 May 2011)] were randomly allocated to two groups (34 lambs in the rape group and 26 lambs in the ryegrass group) and acclimatised to their assigned forages at AgResearch Aorangi Experimental Station. During this initial adaptation period, the lambs in the forage rape group were allowed to graze on growing forage rape in a paddock for 8 h per day in the first two days, for 16 h per day in the second two days, and then continuously after that. During the first four days of adaptation, the lambs spent the remainder of the day grazing a ryegrass dominated pasture. Animals in the ryegrass group grazed on a pure ryegrass sward over the same period. After acclimatisation of 41 days in the paddocks, 24 lambs in the rape group (live weight 40.0 ± 2.1 kg) and 18 lambs in the ryegrass group (40.7 ± 1.6 kg) for the ryegrass group were selected based on health status and live weight change during the outdoor acclimatisation period, and transferred indoors for acclimatisation to the housing conditions.

While indoors, the lambs were fed the same diet as freshly-harvested forage (forage rape or ryegrass) in pens for 3 days and then individually in metabolic crates for 5 days. CH_4_ emissions from the animals were then measured in respiration chambers for two consecutive days. After CH_4_ measurement, 6 animals from each group were selected for measurements of apparent total tract feed digestibility and feed metabolisable energy (ME) content over 7 days. Rumen content samples were taken after feeding on day 46 of the experiment while the animals were in pens for adaptation, and rumen samples were taken before feeding on days 52 or 54 immediately after the animals were removed from the chambers.

After the first period of measurements, the lambs were returned to paddocks of the same forage used during the acclimatisation period. After 38 further days of grazing, the lambs were transferred indoors again and the same treatments and measurements were performed as in the first period. At the beginning of the second indoor experimental period, lambs were 52.8 ± 3.9 kg for the forage rape group and 48.8 ± 2.8 kg for the ryegrass group. In addition, rumen liquid and particulate passage rates were measured in conjunction with the digestibility measurements. Samples for rumen liquid passage rates collected on day 108 were also used for measurement of rumen fermentation products.

During the indoor acclimatisation and experimental periods, the animals were provided fresh forage at a feeding level of 1.5 times their metabolisable energy (ME) maintenance requirement [1]. For this purpose, ME content of the forages was estimated using near infrared reflectance spectroscopy (NIRS; Bruker Optics, model MPA, Ettlingen, Germany). Forage was provided in two equal portions at 0900 and 1600 h. The animals had free access to water at all times.

During the CH_4_ measurement period, fresh forage samples were collected daily and frozen at −20°C for later analysis of nitrate, sulfate, glucosinolate and *S*-methyl L-cysteine sulfoxide (SMCO) concentrations after freeze drying and grinding, and for determination of *in situ* DM degradation kinetics.

**Animal health**

All lambs were dosed with an anthelmintic on day 1 (4 ml product containing 8 mg abamectin, 320 mg Levamisole, 20 mg cobalt and 4 mg selenium; Intervet Schering-Plough Animal Health Ltd., Wellington, New Zealand). Based on analysis of the mineral content of the forages, lambs in the forage rape group were given intramuscular injection with 1.5 ml of iodised peanut oil containing 390 mg of organically bound iodine (Flexidine, Bomac Export Limited, Auckland, New Zealand) and supplied a copper capsule *per os* containing 4 g of CuO on day 3 (Bayer New Zealand Limited, North Shore, New Zealand). These were administered to ensure that lambs had sufficient supply of these elements during the whole experiment, by request of the Animal Ethics Committee. Perennial ryegrass fed lambs were not supplied with CuO to avoid possible toxicity, as ryegrass contains sufficient copper [2]. Although Cu could reduce the number of protozoa in the rumen [3,4] and the number of protozoa is correlated with CH_4_ production [5], Cu supplementation is unlikely to have had an effect on CH_4_ emissions, based on our unpublished *in vitro* data which indicate that both gas and CH_4_ productions were not affected by the addition of CuO at levels of 0, 12.5, 25, 50, 75, 100 and 200 mg/g substrate. To the best of our knowledge, there are no reports on iodine affecting methane emissions.

During the acclimatisation period in the paddock, salt blocks containing 897 mg NaCl, 28 mg Ca, 0.58 mg Zn, 0.25 mg Cu, 0.09 mg I, 0.089 mg Fe, 0.065 mg Co and 0.03 mg Se per g (Dominion Salt Limited, Lake Grassmere, New Zealand) were supplied to both groups. Animals were weighed weekly. All animals were drenched again on day 42 with 9 ml of an anthelmintic compound containing 9 mg abamectin, 360 mg levamisole, and 204 mg oxfendazole (Merial New Zealand Ltd, Auckland, New Zealand), as requested by the Animal Ethics Committee.

Blood samples were taken from the jugular vein on day 46 for determination of methaemoglobin, haemoglobin and haematocrit. The samples for methaemoglobin determination were taken randomly from five lambs in the forage rape group and three in the ryegrass group at 1030 h, 1.5 h after morning feeding, fully filled into blood collection evacuated tubes with lithium heparin anticoagulant (Becton, Dickinson and Company, Franklin Lakes, New Jersey, USA), immediately stored on ice in an insulated container and dispatched for analysis within 1 h after sampling. The analysis was conducted by visible optical spectroscopy by Medlab Central Limited (Palmerston North, New Zealand) using a blood gas analyser (ABL 800 FLEX; Radiometer, Copenhagen, Denmark). Blood samples for haemoglobin and haematocrit determinations were taken at 1430 h from all animals into evacuated blood collection tubes containing EDTA (Becton, Dickinson and Company, Belliver Industrial Estate, Plymouth, UK). The samples were stored at room temperature and analysed on the same day using an automated blood counter (New Zealand Veterinary Pathology Ltd., Palmerston North, New Zealand). In the second period, the blood samples were taken on day 102 of the experiment for methaemoglobin from 6 forage rape-fed and 4 ryegrass-fed lambs at 1030 h and for haemoglobin and haematocrit from all animals at 1430 h.

### Forages

Forage rape (*var*. Titan) and ryegrass (*var*. Ceres One 50 containing endophyte AR1) at the AgResearch Aorangi Experimental Station for animal adaptation were pure swards.

Forage rape sward for the indoor experimental period was established in a paddock (150 m × 20 m) at AgResearch Grasslands Research Centre on 3 March 2011 in Manawatu fine sandy loam soil with a sowing rate of 4.7 kg/ha and di-ammonium phosphate applied at 140 kg/ha.

Perennial ryegrass for the indoor experiments was established in a paddock (120 m × 20 m) at AgResearch Grasslands Research Centre in autumn 2008. Superphosphate (93 g of P kg^-1^, 108 g of S kg^-1^ and 200 g of Ca kg^-1^; Ravensdown Limited, Hornby, New Zealand) was applied at 200 kg ha^-1^ on 12 April 2011 and urea (containing 460 g N kg^-1^) was applied at 60 kg ha^-1^ on 5 May 2011. Before harvest, the paddock was grazed to a residual height of <5 cm on 9 May 2011.

Forage fed to animals during the first (days 42 to 59) and the second (days 98 to 116) indoor experimental periods was harvested daily (1030 to 1200 h) using a sickle bar mower. Both forage rape and ryegrass were in the vegetative state. The forage rape plant height was *ca*. 70 cm and the stem height *ca*. 20 cm in the first period; and *ca*. 68 cm and *ca*. 27 cm, respectively, in the second period, and the stubble height *ca*. 10 cm after cutting. Harvested forage rape contained, on a fresh weight basis, 76% green leaves, 16% stems, 8% dead leaves, and less than 0.5% weeds in the first period, and 66% green leaves, 24% stems, 9% dead leaves, and less than 0.1% weeds in the second period. Ryegrass was harvested at *ca*. 25 cm in height in the first period and *ca*. 34 cm in the second period, with a stubble height at *ca*. 5 cm.

The harvested forage was stored in a cold room (4°C) for the afternoon meal on that day and for the next day’s morning meal. Triplicate samples (*ca*. 150 g fresh weight each) each day were dried at 105^o^C for DM determination, and one sample was dried at 65°C for 48 h for subsequent chemical analysis. Gross energy (GE) content, neutral detergent fibre (NDF), acid detergent fibre (ADF), acid detergent lignin (ADL), crude protein (CP), lipid, ash, hot water-soluble carbohydrate (HWSC) and pectin contents were determined (*n*=3 for each forage and period, 2 from the CH_4_ measurement period, 1 from the digestibility experiment) using the method described in Sun *et al*. [6].

The concentrations of nitrate, sulfur and sulfate (*n*=4 for each forage and period) in forages collected during the CH_4_ measurement periods were determined using methods described by Cataldo *et al*. [7], Araújo *et al*. [8] and Miller [9], respectively. Samples (*n*=6 for Period 1, *n*=4 for Period 2, for each forage) for determination of glucosinolates and SMCO were extracted in hot water and the supernatants used for analysis using HPLC-MS [6].

### Determination of methane and hydrogen emissions

A sheep respiration chamber facility containing three clusters with eight individual chambers each was used to determine CH_4_ and hydrogen (H_2_) emissions. The structure of the chambers was described in detail by Pinares-Patiño *et al*. [10]. The lambs were transferred to chambers in two batches (12 lambs fed forage rape and 9 fed ryegrass in each batch; see Table S1) for the measurements. The measuring procedure was as the same as used by Sun *et al*. [6].

### Measurement of apparent digestibility and metabolisable energy

The protocol for measuring apparent digestibility and ME of the forages was described by Sun *et al*. [6]. The same methods for feed samples were used for the analysis of refusal and faeces. The nitrogen content in the urine samples were determined by the Dumas method (AOAC method 986.06) [11] on a Leco TruSpec CN Carbon/Nitrogen Determinator (Leco Corporation, St. Joseph, Michigan, USA), and energy content determined using a bomb calorimeter (AC 350, Leco Corporation, St. Joseph, Michigan, USA) [12].

### Rumen sampling

Rumen samples (*ca*. 50 ml) were taken from all animals by stomach tubing at 2-3 h after feeding (1030-1130 h) on day 46 and before feeding (0930-1000 h) on days 52-54 of the experiment. An aliquot (1.8 ml) of the sample was collected for volatile fatty acid (VFA) analysis [13], another aliquot (2 ml) was mixed with the same volume of methyl green fixative [14] and stored in dark at 4°C for protozoa counting. The remainder of the samples collected after feeding was stored at -20°C and freeze dried for subsequent analysis of microbial community structure. In the second period of the experiment, on day 102, after feeding, rumen samples were taken from all animals for the analysis of microbial communities. In addition, rumen fluid was sampled using stomach tubing on day 108 at 0, 2, 4, 6, 8, 10, 12 and 24 h after morning feeding for rumen pH and VFA analysis from the 12 sheep which had been randomly selected for rumen passage rate and digestibility measurements, in combination with rumen sampling for determination of liquid passage rate.

### Rumen passage rate determination

Rumen passage rates for liquid and solids were determined in combination with apparent digestibility determination during the second period of the experiment using cobalt ethylene diaminetetraacetic acid (Co-EDTA) as a liquid phase maker and chromium (Cr)-mordanted fibre as a solids marker according to the method of Uden *et al*. [15]. Cr concentrations in faecal samples and Co concentrations in the rumen fluid were analysed using inductively coupled plasma optical emission spectrometry (model Optima 2000 DV; Perkin Elmer, Inc., Waltham, Massachusetts, USA) [16,17].

The change of Co concentration in the rumen samples after dosing was used to calculate the rumen liquid passage rate and the initial Co concentration at 0 h [18] using the package *“nlmrt”* for nonlinear curve fitting in R [19-21]. The rumen liquid pool size was calculated from the amount of Co added to the rumen and the initial Co concentration at 0 h [18]. Cr concentrations in faecal samples collected at specified times after dosing were fitted to a multi-compartment model [22] for estimation of particulate passage rates, using the package *“nlmrt”* for nonlinear curve fitting in R [19-21].

### *In situ* ruminal DM degradation kinetics

Ruminal DM degradation kinetics of freshly-harvested forage rape and ryegrass were evaluated by an *in situ* technique using fistulated cows [23].The samples were collected at days from 50 to 53 and from 105 to 108 during CH_4_ measurements. In both periods, samples (1 kg fresh weight for each forage and day) were taken on four consecutive days to represent 4 field replicates. These samples were frozen and minced for incubation as described in Sun *et al*. [24]. Dacron bags (10 × 10 cm and 50-micron pore size; ANKOM Technology Corporation, Fairport, New York, USA) were filled with 5 g DM of fresh forage and inserted in the rumen of two fistulated Friesian×Jersey dry cows which were fed cut ryegrass pasture at 1.3 times their ME requirements [1]. The cows were fed equal portions twice a day at 0830 and 1630 h. One bag of each forage-period-field replicate was removed from each cow after 2, 4, 6, 8, 9, 10, 11, 12, 13 and 24 h, and two bags after 72 h of incubation using the “all in, all out” procedure of Sun and Waghorn [23]. In total, 16 bags (2 forages × 2 periods × 4 field replicates) were incubated in each of the two cows for the first 10 time points (2-24 h), and 32 bags were used for the 72 h incubation. *In situ* rumen DM degradation kinetics parameters were calculated in the method of Ørskov and McDonald [25] using the NLIN procedure of SAS [26].

### Assessment of the microbial community composition

DNA was extracted from 30 mg of freeze-dried, homogenised rumen contents collected 2-3 h after feeding during Periods 1 and 2 using the PCQI method [27,28]. Bacterial, archaeal and protozoal microbial community compositions were determined as described previously [27]. Sequences over 400 basepairs in length with an average quality score over 25 were retained, resulting in on average 5172 bacterial, 1749 archaeal and 1616 ciliate protozoal reads being available per sample for analysis.

Sequencing data are available in the NCBI Sequence Read Archive, as project PRJNA239421, submission ID “Forage rape”. The sample naming scheme is as follows: “microbial_group.diet.period.animal_ID.sampling_time.sample_ID”.

To estimate microbial numbers, marker loci for bacteria and archaea were enumerated by quantitative PCR in seven samples each of ryegrass and forage rape-fed animals collected after feeding during Period 2, as previously described [29].

### Statistical analyses

Data on forage chemical compositions, nitrate, glucosinolates, SMCO, digestibilities and animal blood profile parameters were analysed with forage and period as experimental factors using the GLM procedure [26] to compare forage rape and ryegrass. CH_4_ emission data were analysed by a linear mixed-effects model with forage and period as fixed effects and measurement batch and chamber cluster as random effects using the MIXED procedure. Rumen fermentation parameters over different sampling times were analysed by a repeated measurements model with compound symmetry correlation structure, and with forage as a fixed effect and animal as a random effect. Rumen passage rates and rumen volume were analysed with forage as an experimental factor using a one way ANOVA procedure. DM degradation parameters were analysed by a mixed effects model with forage, period and their interaction as fixed effects and cow as a random effect, but for fraction *A*, cow was not in the model. The correlation among variables and between two experimental periods in CH_4_ yield was analysed using the CORR procedure [26]. Differences between two forages were considered to be significant at *P*<0.05.

Statistical analyses of rumen microbial data were performed in GenStat for Windows (13th edition, VSN International, Hemel Hempstead, UK, [www.genstat.co.uk](http://www.genstat.co.uk)). Two-way ANOVA (using forage and period as factors) in combination with the Scheffe post-hoc test was used to detect differences in relative abundances of microbial community compositions between forages and periods. Principal coordinate analysis of Bray-Curtis dissimilarity matrices of microbial community composition data was performed in QIIME. Spearman’s Rank correlations were calculated for rumen fermentation and microbial community composition data from Period 1 after feeding. Dependent sample *t*-tests were also used to detect differences in microbial community composition between periods. Phylogenetic groups with an average abundance of <0.5% in all samples were excluded from analyses of microbial community composition data. Protozoal cell density data were analysed using two-way ANOVA with forage and sampling time as factors. qPCR data were compared using *t*-tests, after testing for normality of distribution using the Shapiro-Wilk test. A probability of *P*<0.05 was considered to indicate a significant difference.

**Results**

**Adaptation and animal health**

The lambs were gradually introduced to forage rape and provided Cu and I supplements. The blood parameters (white blood cells, red blood cells, haemoglobin, hematocrit, mean corpuscular volume, mean corpuscular haemoglobin, mean corpuscular hemoglobin concentration, tissue plasminogen activator) examined had smaller values for lambs fed forage rape than for those fed ryegrass, and methaemoglobin concentration was greater (Table S2), but all parameters were within the normal ranges for both forage treatments. No signs of illness or visible abnormal behaviours were observed in both periods of the experiment.

**References for Text S1**

1. Australian Agricultural Council (1990) Feeding standards for Australian livestock: Ruminants. Sydney, NSW, Australia: CSIRO Publications.

2. Grace ND (1994) Managing trace element deficiencies. Palmerston North, New Zealand: Simon Print.

3. Solaiman SG, Craig Jr TJ, Reddy G, Shoemaker CE. Effect of high levels of Cu supplement on growth performance, rumen fermentation, and immune responses in goat kids. Small Rumin Res. 2007;69: 115-123.

4. Kišidayová S, Sviatko P, Zeleňák I. The effect of copper and cobalt supplementation on the rumen ciliate population in sheep. Czech J Anim Sci. 2000;45: 345-348.

5. Guyader J, Eugène M, Nozière P, Morgavi DP, Doreau M, Martin C. Influence of rumen protozoa on methane emission in ruminants: a meta-analysis approach. Animal. 2014;8: 1816-1825.

6. Sun XZ, Waghorn GC, Hoskin SO, Harrison SJ, Muetzel S, Pacheco D. Methane emissions from sheep fed fresh brassicas (*Brassica* spp.) compared to perennial ryegrass (*Lolium perenne*). Anim Feed Sci Technol. 2012;176: 107-116.

7. Cataldo DA, Haroon M, Shroder LE, Younger VL. Rapid colorimetric determination of nitrate in plant tissue by nitration of salicylic acid. Comm Soil Sci Plant Anal. 1975;6: 71-80.

8. Araújo GCL, Gonzalez MH, Ferreira AG, Nogueira ARA, Nóbrega JA. Effect of acid concentration on closed-vessel microwave-assisted digestion of plant materials. Spectrochim Acta Part B At Spectrosc. 2002;57: 2121-2132.

9. Miller RO (1998) Extractable chloride, nitrate, orthophosphate, potassium, and sulfate-sulfur in plant tissue: 2% acetic acid extraction. In: Kalra YP, editor. Handbook of Reference Methods for Plant Analysis. Boca Raton, FL, USA: CRC Press. pp. 115-118.

10. Pinares-Patiño CS, Lassey KR, Martin RJ, Molano G, Fernandez M, MacLean S, et al. Assessment of the sulphur hexafluoride (SF_6_) tracer technique using respiration chambers for estimation of methane emissions from sheep. Anim Feed Sci Technol. 2011;166-167: 201-209.

11. AOAC (2000) Official Method of Analysis, 17th edition. Gaithersburg, MD, USA: Association of Official Analytical Chemists, Inc.

12. Miller DS, Payne PR. A ballistic bomb calorimeter. Br J Nutr. 1959;13: 501-508.

13. Sun XZ, Hoskin SO, Muetzel S, Molano G, Clark H. Effects of forage chicory (*Cichorium intybus*) and perennial ryegrass (*Lolium perenne*) on methane emissions *in vitro* and from sheep. Anim Feed Sci Technol. 2011;166-167: 391-397.

14. Sun XZ, Hoskin SO, Zhang GG, Molano G, Muetzel S, Pinares-Patiño CS, et al. Sheep fed forage chicory (*Cichorium intybus*) or perennial ryegrass (*Lolium perenne*) have similar methane emissions. Anim Feed Sci Technol. 2012;172: 217-225.

15. Udén P, Colucci PE, Van Soest PJ. Investigation of chromium, cerium and cobalt as markers in digesta. Rate of passage studies. J Sci Food Agric. 1980;31: 625-632.

16. Boumans PWJM. ICP: D.c. arc in a new jacket? Spectrochim Acta Part B At Spectrosc. 1980;35: 57-71.

17. Varian Techtron Ltd. (1979) Model 65 Vapor Generation Accessory, Operation Manual. Springvale, Victoria, Australia: Varian Techtron Ltd.

18. Faichney GJ (2005) Digesta flow. In: Dijkstra J, Forbes JM, France J, editors. Quantitative Aspects fo Ruminant Digestion and Metabolism, 2nd edition. Wallingford, UK: CAB International. pp. 49-86.

19. R Core Team (2013) R: A language and environment for statistical computing. R Foundation for Statistical Computing, Vienna, Austria. ISBN 3-900051-07-0. Available: http://www.R-project.org.

20. Nash JC (2012) nlmrt: Functions for nonlinear least squares solutions. R package version 2012-12.16. Available: http://cran.r-project.org/package=nlmrt. Accessed 10 October 2013.

21. Pinheiro J, Bates D, DebRoy S, Sarkar D, the-R-Development-Core-Team (2013) nlme: Linear and nonlinear mixed effects models. R package version 3.1-109. Available: http://cran.r-project.org/web/packages/nlme. Accessed 10 October 2013.

22. Dhanoa MS, Siddons RC, France J, Gale DL. A multicompartmental model to describe marker excretion patterns in ruminant faeces. Br J Nutr. 1985;53: 663-671.

23. Sun XZ, Waghorn GC. Improving *in sacco* incubation technique to evaluate fresh forage for selecting fast-degrading perennial ryegrass (*Lolium perenne* L.). Grass Forage Sci. 2012;67: 437-445.

24. Sun XZ, Waghorn GC, Clark H. Cultivar and age of regrowth effects on physical, chemical and *in sacco* degradation kinetics of vegetative perennial ryegrass (*Lolium perenne* L.). Anim Feed Sci Technol. 2010;155: 172-185.

25. Ørskov ER, McDonald I. The estimation of protein degradability in the rumen from incubation measurements weighed according to rate of passage. J Agric Sci. 1979;92: 499-503.

26. SAS (2003) Statistical Analysis System. User’s Guide: Statistics. Cary, NC, USA: SAS Institute.

27. Rius AG, Kittelmann S, MacDonald KA, Waghorn GC, Janssen PH, Sikkema E. Nitrogen metabolism and rumen microbial enumeration in lactating cows with divergent residual feed intake fed high-digestibility pasture. J Dairy Sci. 2012;95: 5024-5034.

28. Henderson G, Cox F, Kittelmann S, Miri VH, Zethof M, Noel SJ, et al. Effect of DNA extraction methods and sampling techniques on the apparent structure of cow and sheep rumen microbial communities. PLoS One. 2013;8: e74787.

29. Jeyanathan J, Kirs M, Ronimus RS, Hoskin SO, Janssen PH. Methanogen community structure in the rumens of farmed sheep, cattle and red deer fed different diets. FEMS Microbiol Ecol. 2011;76: 311-326.
